# Rapid enteric testing to permit targeted antimicrobial therapy, with and without *Lactobacillus reuteri* probiotics, for paediatric acute diarrhoeal disease in Botswana: A pilot, randomized, factorial, controlled trial

**DOI:** 10.1371/journal.pone.0185177

**Published:** 2017-10-09

**Authors:** Jeffrey M. Pernica, Andrew P. Steenhoff, Margaret Mokomane, Banno Moorad, Kwana Lechiile, Marek Smieja, Loeto Mazhani, Ji Cheng, Matthew S. Kelly, Mark Loeb, Ketil Stordal, David M. Goldfarb

**Affiliations:** 1 Department of Pediatrics, McMaster University, Hamilton, Ontario, Canada; 2 Center for Global Health & Department of Pediatrics, The Children’s Hospital of Philadelphia, Philadelphia, Pennsylvania, United States of America; 3 Botswana-UPenn Partnership, Gaborone, Botswana; 4 Botswana Ministry of Health, National Health Laboratory, Gaborone, Botswana; 5 Department of Pathology and Molecular Medicine, McMaster University, Hamilton, Ontario, Canada; 6 Department of Paediatrics and Adolescent Health, University of Botswana, Gaborone, Botswana; 7 Department of Health Research Methods, Evidence, and Impact, McMaster University, Hamilton, Ontario, Canada; 8 Department of Pediatrics, Duke University, Durham, North Carolina, United States of America; 9 Norwegian Institute of Public Health, Oslo, Norway; 10 Department of Laboratory Medicine, University of British Columbia, Vancouver, British Columbia, Canada; TNO, NETHERLANDS

## Abstract

**Introduction:**

Diarrhoeal disease is the second-leading cause of death in young children. Current guidelines recommend treating children with acute non-bloody diarrhea with oral rehydration solutions and zinc, but not antimicrobials. However, in many resource-limited settings, infections with treatable enteric bacterial and protozoan pathogens are common. Probiotics have shown promise as an adjunct treatment for diarrhoea but have not been studied in sub-Saharan Africa.

**Methods:**

We conducted a pilot, factorial, randomized, placebo-controlled trial of children aged 2–60 months hospitalized in Botswana for acute non-bloody diarrhoea. A rapid test-and-treat intervention, consisting of multiplex PCR testing of rectal swabs taken at enrolment, accompanied by targeted antimicrobial therapy if treatable pathogens were detected, was compared to the reference standard of no stool testing. Additionally, *Lactobacillus reuteri* DSM 17938 x 60 days was compared to placebo treatment. The main objective of this pilot study was to assess feasibility. The primary clinical outcome was the increase in age-standardized height (HAZ) at 60 days adjusted for baseline HAZ.

**Results:**

Seventy-six patients were enrolled over a seven-month study period. We judged that the recruitment rate, lab processing times, communication protocols, provision of specific antimicrobials, and follow-up rates were acceptable. Compared to the reference arm (no stool testing and placebo treatment), the combination of the rapid test-and-treat strategy plus *L*. *reuteri* DSM 17938 was associated with an increase of 0.61 HAZ (95% CI 0.09–1.13) and 93% lower odds of recurrent diarrhoea (OR 0.07, 95%CI 0.01–0.61) at 60 days.

**Discussion:**

We demonstrated that it was feasible to evaluate the study interventions in Botswana. Despite the small sample size, we observed a statistically significant increase in HAZ at 60 days and significantly lower odds of recurrent diarrhoea in children receiving both rapid test-and-treat and *L*. *reuteri*. There is sufficient evidence to warrant proceeding with a larger follow-up trial in a similar setting.

## Introduction

Diarrhoeal disease, also termed gastroenteritis, is the second-leading cause of death worldwide in children less than five years of age [1]. Furthermore, gastrointestinal infection predisposes to the development of severe acute malnutrition (SAM) [2], which itself increases risk of further infections, cognitive maldevelopment, and mortality (from both infectious and noninfectious causes) [3, 4]. New strategies to diminish the impact of diarrhoeal disease in children are sorely needed [5].

Current World Health Organization (WHO) recommendations for the treatment of gastroenteritis involve prompt delivery of rehydration solutions and zinc therapy [6]. Fluid rehydration is of prime importance regardless of the aetiology of infection to reverse dehydration and treat hypovolaemic shock. The provision of antimicrobials is recommended only if blood is observed in the stools; this recommendation is probably linked to the assumption that the majority of children presenting with acute non-bloody diarrhoea are infected with viral pathogens, for whom no specific treatment is available. However, we have previously found that up to one-third of children in Botswana admitted to hospital without dysentery harboured a potentially treatable pathogen in their stool [7], and other investigators have observed similar findings in other low- and middle-income countries [8, 9]. Although it is not possible to reliably distinguish treatable bacterial or protozoal gastroenteritis from non-treatable gastroenteritis on clinical grounds in the absence of bloody stools, the use of rapid enteric diagnostics at the time of patient presentation could permit the use of targeted antimicrobial treatment. Some have suggested that timely antimicrobial use in children with treatable gastroenteritis could improve symptoms and prevent subsequent growth deficits and/or mortality [10].

There has been substantial interest in the use of probiotics as adjunctive therapy for children with acute gastroenteritis, predominantly in the context of viral infection. Numerous clinical trials have been conducted, predominantly in upper-income countries [11–16]; a systematic review of 3 randomized treatment trials done in Turkey and Italy showed that *Lactobacillus*. *reuteri* (a commensal of the human gastrointestinal tract) administration, as compared to placebo or no intervention, reduced the duration of diarrhoea by 24.8 hours (95% CI 38.8 hours to 10.8 hours) [17]. However, no trials have been conducted in sub-Saharan Africa, where the burden of diarrhoeal disease is very high and both mortality and morbidity are much more substantial, due in part to co-existing malnutrition and/or environmental enteropathy. Additionally, there are almost no data on the use of probiotics for the adjunctive treatment of bacterial or protozoan gastroenteritis.

We hypothesized that rapid diagnostic testing followed by specific targeted antimicrobial therapy, alone or in combination with *L*. *reuteri* DSM 17938, would improve linear growth and reduce other morbidity and mortality, in children admitted to hospital because of gastroenteritis in a high-burden setting. We chose linear growth as a primary outcome because of its close association with overall health and cognitive development [18, 19]. In order to investigate these interventions simultaneously, a factorial randomized controlled trial was designed. To allow us to explore the practicality of operationalizing these interventions in Botswana and ascertain the variance of treatment effects, we undertook a pilot trial that would determine the later feasibility of a formal, adequately-powered randomized controlled trial.

## Methods

### Study design and participants

A pilot, multi-centre, randomized, controlled, factorial, clinical trial was conducted at Princess Marina Hospital (Gaborone, Botswana) from March-December 2014; Bamalete Lutheran Hospital (Ramotswa) and Scottish Livingstone Hospital (Molepolole) were added as study sites in September 2014. Children aged 2 to 60 months admitted to the paediatric wards of the study hospitals because of acute diarrhoeal disease were eligible. (The original inclusion criteria stated 3–60 months; the relevant amendment was submitted prior to commencement of enrolment.) ‘Diarrhoeal disease’ was defined as the passage of at least 3 watery stools in a single 24-hour period preceding hospitalization; ‘acute’ referred to illness less than 14 days in duration. Children with HIV infection/exposure were not excluded. Children with severe acute malnutrition (SAM), defined as weight less than 3 standard deviations (SD) below the mean, or weight-for-length less than 3 SD below the mean, were eligible to participate in the study but were not randomized to one of the interventions (*Lactobacillus reuteri* DSM 17938 or placebo, see below). Their outcomes were analysed separately, owing to the fact that these children’s baseline prognosis was substantially worse than children without malnutrition. Children with the following conditions were excluded: visibly bloody diarrhoea by history or at presentation, medical comorbidities (ie. inflammatory bowel disease, malignancy, cystic fibrosis), suspected bacterial sepsis or meningitis leading to the initiation of a 3^rd^ generation cephalosporin, suspected primary urinary tract infection and a urinalysis with positive leukocytes or nitrites, or suspected primary pneumonia with a chest radiograph showing a focal consolidative process. Additionally, children were excluded if they had a household member with an identified bacterial or parasitic enteric infection, were transferred from another health facility with treatment with broad-spectrum antimicrobial therapy already begun (ie. cefotaxime, nalidixic acid, or ciprofloxacin), or developed nosocomial diarrhoea in hospital. Children living outside the hospitals’ catchment areas were excluded, as were those whose caregivers did not have access to a telephone (either landline or mobile), or those without a fixed address.

Written informed consent was completed by all caregivers. The trial protocol was approved by the research ethics boards of the Botswana Health Research and Development Committee, the Princess Marina Hospital, the University of Pennsylvania, USA, and McMaster University, Canada. The trial conduct complied with the principles of the International Conference on Harmonisation guidelines for Good Clinical Practice (GCP). The trial was registered on clinicaltrials.gov (NCT 02025452).

### Randomization and masking

Within 48 hours of admission to hospital, potential participants and caregivers were contacted on the paediatric ward by the research nurse. If consent was granted, children without SAM were randomized using a web-based system (Research Electronic Data Capture, REDCap) [20] to one of four treatment arms in a 1:1:1:1 ratio:

1. Rapid enteric testing, followed by targeted antimicrobial therapy if a treatable pathogen was detected, plus *Lactobacillus reuteri* DSM 17938 5x10^8^ colony-forming units (cfu) once daily x 60 days
2. Rapid enteric testing, followed by targeted antimicrobial therapy if a treatable pathogen was detected, plus placebo daily x 60 days
3. Standard care (no rapid enteric testing, so no targeted antimicrobial therapy as per WHO guidelines) plus *Lactobacillus reuteri* DSM 17938 5x10^8^ cfu once daily x 60 days
4. Standard care (no rapid enteric testing, so no targeted antimicrobial therapy as per WHO guidelines) plus placebo daily x 60 days.

Children with SAM were treated differently and randomized to rapid enteric testing or standard care in a 1:1 ratio (i.e., were not randomized to *L*. *reuteri* or placebo) using a separate REDCap program. All study personnel and participants were aware of whether rapid enteric diagnostics were conducted or not. Conversely, all study personnel and participants were blinded to allocation with *L*. *reuteri* or placebo, which were contained in identical-appearing bottles. The randomization sequence was prepared by the study statistician using a random number generator.

### Procedures

After informed consent was granted, and eligibility verified, information was collected by the research nurse relating to the demographics and socioeconomic status of the child and family, details of the illness prompting admission to hospital, HIV exposure/infection status, immunization history, anthropometrics, and treatment received for the index hospitalization (including fluid rehydration and zinc therapy). The Vesikari score, an index of gastroenteritis severity [21], was calculated for all participants. Height, weight, and middle upper arm circumference were measured using standard protocols and source documentation was entered on paper forms, which were kept in a secure office. Study data were later entered and managed using REDCap electronic data capture tools hosted at McMaster University. A flocked rectal swab (FecalSwab, Copan Italia S.A., Brescia, Italy) and a bulk stool sample were collected for each participant.

Participants randomized to rapid enteric testing had enteric specimens (rectal swab and bulk stool) processed at the National Health Laboratory on the day of enrolment. It was recommended by the study team that participants found to have stool positive for *Campylobacter*, *Shigella*, or enterotoxigenic *E*. *coli* (ETEC) be treated with azithromycin 10 mg/kg/day for 3 days and that participants found to have stool positive for *Cryptosporidium* be treated with nitazoxanide (7.5 mg/kg to maximum of 100 mg PO BID for ages 5–11 months, 100 mg PO BID for ages 1–3 years, and 200 mg PO BID for ages 4+ years, all for 3 days). The research nurse brought testing results and treatment recommendations to the clinical team on the day of enrolment (or as soon as possible thereafter), who made the final decision about the provision of antimicrobials; azithromycin and nitazoxanide were provided free of charge to participants. If participants’ stool was positive for *Salmonella* or *Giardia*, this information was relayed to the local clinical medical team without specific treatment recommendations.

The probiotic or placebo was started on the day of enrolment. *L*. *reuteri* DSM 17938 was suspended in vegetable oil and provided in two small dark amber bottles with a dropper tip; the dose was 5 drops by mouth (5x10^8^ cfu) daily. The placebo consisted of the vegetable oil vehicle dispensed in identical bottles. Probiotics and placebo were manufactured and provided by BioGaia A.B. (Stockholm, Sweden). All antimicrobials and probiotics were kept refrigerated while the child was in hospital and all families were advised to maintain refrigeration after discharge, if possible. Participants were followed daily in hospital so that adherence to antimicrobials and probiotic/placebo could be verified by the study team. The decision to use *L*. *reuteri* was because of previous studies suggesting effectiveness in preventing diarrhoea [22] and promoting growth [23]; a 60-day treatment course was selected to maximize effectiveness.

### Laboratory testing

Flocked swabs (Copan Italia, Brescia, Italy) were used to collect rectal specimens; these were immediately pre-treated as previously described [24]. Total nucleic acid extraction was performed on the MagNA Pure instrument (Roche Diagnostics, Basel, Switzerland) at the National Health Laboratory according to manufacturer instructions.

Two previously described [24, 25] laboratory-developed real time multiplex PCR assays were performed using the ABI 7500 FAST (Life Technologies, Carlsbad, CA) detected the following gastrointestinal pathogens: *Shigella*, *Salmonella*, *Campylobacter*, ETEC LT/ST, *Giardia* and *Cryptosporidium*. Briefly, five microlitres of extracted nucleic acid from each swab were added to the primers, probes, and the QuantiTect multiplex no ROX PCR kit reagents for amplification. Extraction and master-mix negative controls and a positive control were included with each assay. Cycling parameters were: 1 min at 60°C, 15 min at 95°C, followed by 45 cycles of 20 sec at 95°C and 1 min 10 sec at 60°C, and a final hold of 1min at 60°C. At the start of the trial, prior to implementation of the *Cryptosporidium* PCR assay, a commercial antigen detection test (ImmunoCard STAT^®^, Meridian Bioscience) was utilized on bulk stool samples according to manufacturer instructions.

Stool samples collected at 60 day follow up were stored at -80C within 24 hours of collection at the National Health Laboratory. At the end of the study, all stool samples were shipped to McMaster University, Canada. Commercial immunoassays were used to detect and quantify neopterin (Genway Biotech, San Diego, CA), alpha-1-antitrypsin (BioVendor, Asheville, NC), and myeloperoxidase (Affinity Diagnostics, Toronto, Canada) in the stools from the 60-day followup visit. Results from these assays were used to generate the environmental enteropathy score (EES) as described previously [26].

### Feasibility outcomes

The feasibility outcomes of the trial were the following: the number of participants enrolled within 12 months (projected: 100); the proportion of participants having rectal swab specimens obtained within 18 hours of admission (projected: 95%); the proportion of rectal swabs in the rapid-test arms having results within 48 hours of admission (projected: 95%); the proportion of participants in the rapid-test arms found to have a treatable enteric pathogen being prescribed appropriate antimicrobials (projected: 95%); the proportion of participants prescribed antimicrobials that start these medications within 24 hours of the test being resulted (projected: 95%); the proportion of participants contacted within 7–14 days after discharge from hospital (projected: 95%); and the proportion of participants contacted 60 days after enrolment (projected: 90%). Feasibility outcomes were reported for all participants.

### Clinical outcomes

The primary clinical outcome was height-for-age (z-score, HAZ) at 60 days, adjusted for baseline HAZ. Secondary outcomes included 7-day mortality, 60-day mortality, recurrence of diarrhoea in the 60-day followup period, presence of stunting (HAZ<-2) at 60 days, weight-for-age (z-score, WAZ) at 60 days adjusted for baseline WAZ, length of stay in hospital, number of days of fever in hospital, number of days of 3+ watery stools in hospital, and environmental enteropathy score (EES) at 60 days. (The original primary outcome was 60-day mortality, but due to the rarity of this outcome, the relevant amendment was submitted to alter this prior to the commencement of enrolment.) Participants were asked to come back to the hospital for repeat anthropometry and to measure other secondary outcomes; if they were not able to do so, they were visited at home by the research nurse. Volume of probiotic/placebo remaining in dispensed bottles was measured to assess adherence. If participants had taken the probiotic/placebo with perfect adherence, one would have expected approximately 240 drops remaining, as bottles contained approximately 270 drops (10 mL). (‘Perfect’ adherence in terms of drops conserved was not a study goal, as drops were often expended during training of the caregivers in addition to the routine administration to participants.)

### Statistical analysis

The study flow, including screening, consenting, randomizing, following-up and final analysis, followed the CONSORT Guideline (http://www.consort-statement.org).for conducting and reporting RCTs (S1 File, CONSORT checklist).

Proportions were used to report the feasibility outcomes for recruitment and follow-up rates. Patient demographic characteristic and baseline information were reported by the intervention arms. Categorical and continuous variables were reported respectively as count (%) or mean (standard deviation (SD)) /median (1^st^ quartile (Q1), 3^rd^ quartile (Q3)). The clinical outcomes were reported for each treatment arms. For continuous outcomes, mean (SD) / median (Q1, Q3) were used where they were appropriate; for dichotomous outcomes, count (%) were used. The differences of the clinical outcomes evaluated using linear (for continuous outcomes) or logistic (for dichotomous outcomes) regression, and the results were reported respectively as β coefficients or odds ratios with the corresponding 95% confidence intervals (CIs). Given the difference in prognosis between children with and without SAM, these populations were intended to be analyzed separately. All statistical analyses were conducted using SAS 9.3 (SAS, Cary, NC) or Stata 13 (StataCorp, College Station, TX).

For this pilot study, where the primary objective was to establish feasibility (rather than to demonstrate effectiveness), no formal sample size calculation was performed.

### Role of the funding sources

The main sponsor, Grand Challenges Canada, had no role in the study design, data collection, data analysis or interpretation, or writing of the report. The *L*. *reuteri* DSM 17938 preparations and matching placebos were donated by BioGaia A.B. and the flocked swabs were provided by Copan Italia S.A. Neither of these entities had any role in the study design, data collection, data analysis or interpretation, or writing of the report.

## Results

**Fig 1:**
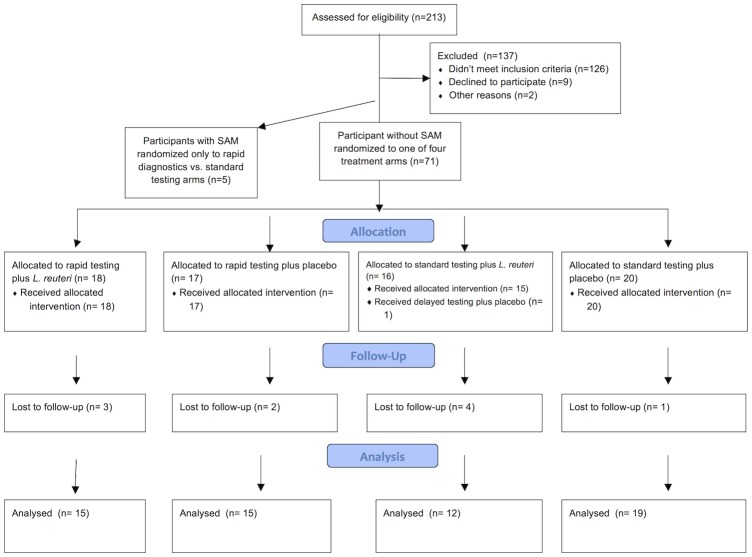
CONSORT diagram.

Recruitment was active at Princess Marina Hospital from 10 March to 30 September 2014 and at Bamalete Lutheran Hospital and Scottish Livingstone Hospital from 1 August to 30 September 2014. During the study period, a total of 213 children hospitalized with diarrhoea were screened for eligibility. 76 participants were enrolled– 5 with SAM and 71 without (Fig 1).

Table 1 describes the baseline characteristics of the 71 study participants without SAM.

**Table 1:** Baseline group characteristics for participants without severe acute malnutrition (n = 71).

	Rapid testing and placebo	Rapid testing and *L*. *reuteri*	Delayed testing and placebo	Delayed testing and *L*. *reuteri*
	(n = 17)	(n = 18)	(n = 20)	(n = 16)
Age (year): mean (SD)	1.11 (0.76)	0.98 (0.71)	1.21 (0.89)	1.07 (0.49)
Female gender; count (%)	9 (52.9)	5 (27.8)	9 (45.0)	9 (56.3)
Weight-for-age Z score; mean (SD)	-1.2 (1.1)	-0.9 (1.0)	-0.7 (1.3)	-0.6 (1.9)
Height-for-age Z score; mean (SD)	-0.6 (2.0)	-0.8 (1.4)	-0.4 (1.4)	-0.4 (2.1)
Child middle upper arm circumference (MUAC) (cm); mean (SD)	12.1 (2.9)	13.2 (1.1)	13.7 (1.6)	14.3 (2.0)
Number of days of diarrhoea prior to admission; median (Q1,Q3)	1 (1, 3)	3 (2, 3)	3 (2, 4.5)	2 (1, 4)
Maximum number of diarrhoea episodes in a 24-hour period; median (Q1,Q3)	6 (6, 8)	6 (5, 8)	6 (5, 7)	4.5 (4, 7.5)
Number of days of vomiting prior to admission; median (Q1,Q3)	5 (4, 6)	4 (2, 5)	5 (3, 7)	4 (2, 5)
Maximum number of vomiting episodes in a 24-hour period; median (Q1,Q3)	1 (1 4)	2 (1, 3)	2 (1, 4)	2.5 (1, 4)
Oral rehydration solution given prior to hospitalization; count (%)	13 (76.5)	8 (44.4)	16 (80.0)	13 (81.3)
Fever at admission; count (%)	15 (88.2)	12 (66.7)	12 (60.0)	13 (81.3)
Rehydration provided intravenously in hospital; count (%)	14 (82.3)	15 (83.3)	20 (100)	13 (81.2)
Child’s Vesikari score; mean (SD)	12.8(2.6)	11.9 (2.7)	12.8 (3.0)	12.9 (2.1)
HIV-infected or HIV-exposed with infection status unconfirmed; count (%)	2 (11.7)	4 (22.2)	2 (10.0)	0 (0.0)
HIV-exposed but known uninfected; count (%)	2 (11.8)	6 (33.3)	4 (20.0)	5 (31.3)
HIV-unexposed; count (%)	11 (64.7)	7 (38.9)	14 (70.0)	7 (43.8)
More than 2 children under age of 5 living in the same household^1^; count (%)	3 (17.7)	4 (22.2)	3 (15.0)	0 (0.0)
Participant’s mother married; count (%)	2 (11.7)	2 (11.1)	1 (5.0)	2
Mother has less than a full secondary school education; count (%)	3 (17.6)	3 (16.7)	1 (5.0)	2 (12.5)
No electricity in the household; count (%)	4 (23.5)	4 (22.2)	10 (52.6)	6 (37.5)
No refrigerator in house^2^; count (%)	6 (35.3)	4(22.2)	7 (35.0)	6 (37.5)
Family uses a shared community tap for water; count (%)	12 (70.6)	9 (50.0)	15 (75.0)	11 (68.8)

^1^Including the index child.

^2^Some homes have appliances without electricity by way of generators.

SD, standard deviation; Q, quartile; MUAC, middle upper arm circumference; IV, intravenous

Many study participants had pathogens detected in their enteric specimens. Treatable pathogens found in participants randomized to the rapid-testing arm, and those found after study close in participants randomized to the delayed-testing arm, are listed in Table 2.

**Table 2:** Enteropathogens detected in study participants, stratified by testing arm.

	# Participants receiving rapid testing who had stools positive for the listed pathogen	# Participants receiving standard testing who had stools positive for the listed pathogen
*Campylobacter*	10 (26%)	15 (42%)
*Shigella*	7 (18%)	8 (22%)
ETEC	4 (11%)	4 (11%)
*Cryptosporidium*	2 (5%)	4 (11%)
any of the above	19 (50%)	20 (56%)

### Feasibility outcomes

Some, but not all, of the feasibility outcomes were achieved. Only 76 participants were enrolled. There were some errors in enrolment and randomization: 3 children were incorrectly included (one age-ineligible and two were enrolled > 48 hours after hospitalization), 2 participants received the incorrect treatment allocation after randomization (one of whom was ineligible), and there were 2 other randomizations that were never assigned to participants. The median time between hospitalization and rectal swab delivery to the laboratory was 19.1 hours (25–75%ile 12.8–27.0 hours), with 55% of the participants’ specimens delivered to the laboratory >18 hours after admission. Sixty-five percent of rectal swabs in the rapid treatment groups were processed with results provided to clinicians within 24 hours of enrolment, at a median of 6.9 hours after admission. There was a biphasic distribution of result reporting times, with 61% of participants having results available to the clinician on the day of enrolment, and 29% of participants having results available on the day after enrolment. Only three participants had test reports provided after the first post-enrolment day; of these, only one was found to harbor a treatable enteric pathogen. For all participants receiving rapid testing found to have treatable pathogens in stool specimens (n = 19), azithromycin and/or nitazoxanide were started on the day of enrolment in 12 (63%) of participants, and on the day after enrolment in 6 (32%). All participants began taking appropriate antimicrobials on the same day that testing reports were provided, after a median of 20 minutes (Q1-Q3 2–75 minutes). Only 47 (63%) participants in total were contacted at 7–14 days post-enrolment, but 68 (91%) participants were successfully followed-up at 60 days for the measurement of the primary outcome.

The median length of stay in hospital was 3 days (Q1-Q3 2–7 days). Caregivers of 4 participants (5%) changed their minds subsequent to recruitment and decided not to give their children the probiotic or placebo treatments (2 in each group); no other major problems with adherence were reported by the caregivers. These children were still followed-up at 60 days.

In addition to these pre-specified feasibility outcomes, we obtained data on adherence. Caregivers of 33 participants returned the probiotic/placebo bottles to the investigators; the median number of drops remaining was 153 (25–75%ile 27–289 drops).

### Clinical outcomes

We analysed outcomes for children with and without SAM separately. Table 3 presents outcomes for participants without SAM. Given that there were only five children with SAM enrolled in the study, an analysis of their clinical outcomes will not be presented in this report.

**Table 3:** Feasibility outcomes achieved.

Feasibility outcome	Initial target	Result achieved	Comment
Enrolment (12-month period)	100	76	Study period limited to 7 months.
Proportion of swabs obtained <18 h after admission	95%	45%	Inclusion criteria later changed to allow enrolments within 48 hours after admission.
Proportion of swabs resulted <48 h after admission	95%	81%	Inclusion criteria later changed to allow enrolments within 48 hours after admission.
Proportion of participants in rapid-test arm found to have a treatable enteric pathogen being prescribed antimicrobials	95%	100%	
Proportion of participants prescribed antimicrobials that start medications <24 h after test result	95%	100%	
Proportion of participants contacted within 7–14 days after discharge	95%	63%	
Proportion of participants followed-up at 60 days	90%	91%	

Key study outcomes, relative to the reference standard-therapy arm of no rapid testing and placebo (i.e., no study interventions), with all anthropometry adjusted for baseline measures, are presented in Table 4. We did not find any statistically significant differences in HAZ, WAZ, odds of recurrent diarrhoea, or EE scores in participants receiving rapid test-and-treat and placebo compared to the reference group. However, we observed an increase of 0.33 in HAZ at 60 days, a more moderate increase of 0.17 in WAZ at 60 days, a decrease in the odds of recurrent diarrhea in the 60-day followup period by 0.55, and an improvement in EE score by 1.08; none of these were statistically significant at the nominal 0.05 level. The combination of both rapid test-and-treat and *L*. *reuteri* therapy was associated with a statistically significant increase of 0.61 in HAZ at 60 days and statistically lower odds of recurrent diarrhoea in the followup period (OR 0.07); though an increase of 0.20 in WAZ and an improvement 1.25 in EE at 60 days were observed, neither of these were statistically significant. The use of *L*. *reuteri* by itself (i.e., without rapid test-and-treat) was associated with statistically lower odds of recurrent diarrhoea in the 60-day follow-up period (OR 0.10) and a non-significant increase of 0.51 in HAZ at 60 days.

**Table 4:** Comparison of outcomes between treatment arms.

Groups	HAZ at 60 days after adjusting for baseline	WAZ at 60 days after adjusting for baseline	Recurrent diarrhea	EE Score
Reference group: Delayed testing + placebo	Difference (95% CI)	Difference (95% CI)	Odds Ratio (95% CI)	Difference (95% CI)
Rapid testing + placebo Delayed testing + *L*. *reuteri* Rapid testing + *L*. *reuteri*	0.33 (-0.24, 0.89) 0.51 (-0.08, 1.11) **0.61 (0.09, 1.13)**	0.17 (-0.31, 0.65) -0.03 (-0.56, 0.48) 0.20 (-0.25, 0.66)	0.45 (0.11, 1.79) **0.10 (0.01, 0.93)** **0.07 (0.01, 0.61)**	-1.08 (-2.56, 0.40) -0.55 (-2.00, 0.90) -1.25 (-2.93, 0.43)

Bold figures indicate statistical significance with *p*<0.05.

We did not observe any serious adverse events, including death, in any of the study participants during the study period.

## Discussion

The primary finding of our study was that rapid test-and-treat algorithms to manage severe acute diarrhoea are feasible in resource-limited settings, including sub-Saharan Africa. We experienced few recruitment difficulties, probably owing to the fact that both caregivers and clinicians were receptive to the interventions under study and thus motivated to participate, and there was minimal loss to follow up. In addition, we demonstrated the practicality of obtaining enteric specimens in a timely way through the use of rectal swabs, coordinating multiplex PCR testing in a local laboratory, and delivering these results promptly to the participants’ physicians to facilitate the initiation of antimicrobial therapy within a clinically relevant interval. Despite the fact that acute diarrhoeal disease causes a staggering amount of mortality and morbidity in young children globally [27], and bacterial and protozoan enteropathogens are common causes of paediatric gastroenteritis in resource-limited settings [8, 9, 28, 29], we know of no other randomized trials that have evaluated the benefits of rapid test-and-treat algorithms to manage acute diarrhoea in low- or middle-income countries. We are also unaware of any randomized trials evaluating the effectiveness of probiotic preparations as treatment for acute gastroenteritis in sub-Saharan Africa.

The trial did not meet all pre-specified feasibility outcomes. Only 76 participants were enrolled; however, the overall study period was limited to seven months rather than the intended twelve. More than half of the study participants’ swabs were delivered to the testing laboratory greater than 18 hours after admission, falling short of our expectation. It should be noted, however, that we had originally planned to enroll children only within 24 hours of hospitalization; subsequent protocol modifications permitted the recruitment of children < 48 hours after admission. Though we were not able to provide 85% of participants with test results within 24 hours of enrolment, results were still available within a clinically relevant time-frame, and only one child had treatment delayed past the first post-enrolment day. Despite not all feasibility outcomes having been achieved, we feel that a follow-up trial will be possible in southern Botswana, especially if faster next-generation assays were available; more reliable diagnostics with shorter turn-around times would probably maximize the number of participants receiving actionable test results on the day of enrolment.

The clinical outcomes observed were encouraging and deserve further study in a larger, adequately powered trial. As noted before, the combination of rapid diagnostics/targeted therapy and *L*. *reuteri* treatment was associated with a considerable decrease (OR 0.07) in recurrent diarrhoea as well as a dramatic 0.61 Z increase in standardized height (adjusted for baseline) at 60 days, both statistically significant outcomes. Furthermore, there were encouraging signs that the rapid test-and-treat approach alone (ie. in combination with placebo) may also lead to clinically relevant increases in height and decreases in recurrent diarrhoea. Our results are consistent with a meta-analysis of RCTs that observed a small increase in height associated with antibiotic therapy in children living in resource-limited settings [30]; we would hypothesize that this benefit may have been mediated through the treatment of clinical or subclinical intestinal infections, lending support for our approach. It may well be that the decrease in recurrent diarrhoea was an important factor contributing to height preservation in this cohort, though the study design precludes any definitive statements about mechanism of action of the interventions under study.

One of the major strengths of the study design is the relevance of the primary clinical outcome measure, which makes it easier for relevant stakeholders to appreciate the benefits of the interventions of interest. Key limitations include the small size of this pilot study and lack of statistical power, which precludes making definitive conclusions about the magnitude of effect associated with the interventions of interest, given how frequently exaggerated estimates of effect have been found in small studies [31]. There were randomization issues that were primarily due to internet connectivity issues. Furthermore, we were unable to recruit a large number of participants with severe acute malnutrition in this pilot study. As these are the children who are the most vulnerable and the most likely to experience poor outcomes, they may have the most to gain from these interventions, and so including them in greater numbers in future studies will be crucial.

The theory behind rapid test-and-treat methodology is simple: providing antimicrobials to children with severe gastroenteritis caused by treatable pathogens can reduce associated morbidity and mortality by 52–99% [10, 32]. Indeed, many travel medicine guidelines recommend providing broad-spectrum antibacterials to travellers visiting resource-limited countries *in the event* that they develop diarrhoea, even without confirmation of infection with a treatable bacterial or protozoan pathogen [33, 34]. Children from resource-limited settings are at higher risk of longer-term neurodevelopmental sequelae or fatal outcomes resulting from diarrhoeal disease [7], which provides a rationale for empiric antibiotics for acute diarrhoea in high-burden settings [35]. Resistance to the broad-spectrum antimicrobials in use currently for the treatment of many bacterial enteropathogens is a concern, so a diagnostic-driven strategy is a reasonable compromise between the unacceptable mortality and morbidity associated with untreated bacterial/protozoan gastroenteritis and the widespread development of drug resistance that could result from universal antimicrobial treatment.

The mechanisms by which probiotics can diminish the impact of enteric infections are unknown, but may include competition with enteric pathogens for binding sites and nutrients within the intestine, modification of the composition of the intestinal microbiome, stimulation of the host immune response, and/or improvement of intestinal barrier function [36, 37]. It seems logical to suppose that the main mechanism of probiotic action is though effects on the gut microbiome; a previous RCT did not find significant differences between the intestinal microbiota of participants who received *L*. *reuteri* DSM 17938 and those that received placebo, but only 29 infants were enrolled and analysis was restricted to 16S rRNA profiling [38]. Probiotics’ beneficial effects may be mediated through the formation of biofilms in the intestinal environment, as *Lactobacillus* species have been observed to do; biofilms formed by *L*. *reuteri* strains related to DSM 17938 have been shown to produce large quantities of reuterin, a molecule that can inhibit the growth of many different putative intestinal pathogens [39]. Our study is the first randomized trial to our knowledge that has assessed the effect of probiotic therapy in children with gastroenteritis in sub-Saharan Africa, and the results suggest that there may be benefit associated with probiotic treatment of severe gastroenteritis in children. We provided probiotic therapy for a full 60 days; in practice, it may be difficult to ensure adherence to this long of a regimen. Further work needs to be done to establish the true magnitude of effect associated with *L*. *reuteri* supplementation, as well as how much the treatment course can be shortened without losing benefit [22].

In conclusion, we believe a large, adequately powered randomized controlled trial is warranted, to further gauge the impact of rapid diagnostic testing and/or the use of *L*. *reuteri* DSM 17938 for the treatment of acute diarrhoeal disease in children hospitalized with diarrhoea in Botswana.

## Supporting information

S1 File CONSORT checklist. CONSORT checklist.
(DOCX)

S2 File Protocol. Original trial protocol.
(DOCX)

## References

[1] GBD Mortality and Causes of Death Collaborators. Global, regional, and national life expectancy, all-cause mortality, and cause-specific mortality for 249 causes of death, 1980–2015: a systematic analysis for the Global Burden of Disease Study 2015. Lancet. 2016;388(10053):1459–544. doi: 10.1016/S0140-6736(16)31012-1 . 27733281 10.1016/S0140-6736(16)31012-1 PMC5388903

[2] Mata LJ, Urrutia JJ, Albertazzi C, Pellecer O, Arellano E. Influence of recurrent infections on nutrition and growth of children in Guatemala. The American journal of clinical nutrition. 1972;25(11):1267–75. . 5086049 10.1093/ajcn/25.11.1267

[3] Scrimshaw NS. Historical concepts of interactions, synergism and antagonism between nutrition and infection. The Journal of nutrition. 2003;133(1):316S–21S. . 12514318 10.1093/jn/133.1.316S

[4] Collins S, Dent N, Binns P, Bahwere P, Sadler K, Hallam A. Management of severe acute malnutrition in children. Lancet. 2006;368(9551):1992–2000. doi: 10.1016/S0140-6736(06)69443-9 . 17141707 10.1016/S0140-6736(06)69443-9

[5] Bhutta ZA, Black RE. Global maternal, newborn, and child health—so near and yet so far. The New England journal of medicine. 2013;369(23):2226–35. doi: 10.1056/NEJMra1111853 . 24304052 10.1056/NEJMra1111853

[6] The treatment of diarrhoea; a manual for physicians and other senior health workers Geneva: World Health Organization; 2005 [cited 2016 September 19]. http://apps.who.int/iris/bitstream/10665/43209/1/9241593180.pdf.

[7] Pernica JM, Steenhoff AP, Welch H, Mokomane M, Quaye I, Arscott-Mills T, et al Correlation of Clinical Outcomes With Multiplex Molecular Testing of Stool From Children Admitted to Hospital With Gastroenteritis in Botswana. J Pediatric Infect Dis Soc. 2016;5(3):312–8. doi: 10.1093/jpids/piv028 . 26407262 10.1093/jpids/piv028 PMC5125452

[8] Taniuchi M, Sobuz SU, Begum S, Platts-Mills JA, Liu J, Yang Z, et al Etiology of diarrhea in Bangladeshi infants in the first year of life analyzed using molecular methods. The Journal of infectious diseases. 2013;208(11):1794–802. Epub 2013/09/18. doi: 10.1093/infdis/jit507 . 24041797 10.1093/infdis/jit507 PMC3814844

[9] Pavlinac PB, Denno DM, John-Stewart GC, Onchiri FM, Naulikha JM, Odundo EA, et al Failure of syndrome-based diarrhea management guidelines to detect Shigella infections in Kenyan children. Journal of the Pediatric Infectious Disease Society. 2015 Epub 12 July 2015. doi: 10.1093/jpids/piv037 26407270 10.1093/jpids/piv037 PMC5181358

[10] Salam RA, Das JK, Bhutta ZA. Current issues and priorities in childhood nutrition, growth, and infections. The Journal of nutrition. 2015;145(5):1116S–22S. doi: 10.3945/jn.114.194720 . 25833888 10.3945/jn.114.194720 PMC4410495

[11] Allen SJ, Martinez EG, Gregorio GV, Dans LF. Probiotics for treating acute infectious diarrhoea. Cochrane Database Syst Rev. 2010; (11):CD003048 doi: 10.1002/14651858.CD003048.pub3 . 21069673 10.1002/14651858.CD003048.pub3 PMC6532699

[12] Freedman SB, Pasichnyk D, Black KJ, Fitzpatrick E, Gouin S, Milne A, et al Gastroenteritis Therapies in Developed Countries: Systematic Review and Meta-Analysis. PloS one. 2015;10(6):e0128754 doi: 10.1371/journal.pone.0128754 . 26075617 10.1371/journal.pone.0128754 PMC4468143

[13] Freedman SB, Sherman PM, Willan A, Johnson D, Gouin S, Schuh S, et al Emergency Department Treatment of Children With Diarrhea Who Attend Day Care: A Randomized Multidose Trial of a Lactobacillus helveticus and Lactobacillus rhamnosus Combination Probiotic. Clinical pediatrics. 2015;54(12):1158–66. doi: 10.1177/0009922815569200 . 25669920 10.1177/0009922815569200

[14] Schnadower D, Finkelstein Y, Freedman SB. Ondansetron and probiotics in the management of pediatric acute gastroenteritis in developed countries. Curr Opin Gastroenterol. 2015;31(1):1–6. doi: 10.1097/MOG.0000000000000132 . 25333367 10.1097/MOG.0000000000000132

[15] Freedman SB, Williamson-Urquhart S, Schuh S, Sherman PM, Farion KJ, Gouin S, et al Impact of emergency department probiotic treatment of pediatric gastroenteritis: study protocol for the PROGUT (Probiotic Regimen for Outpatient Gastroenteritis Utility of Treatment) randomized controlled trial. Trials. 2014;15:170 doi: 10.1186/1745-6215-15-170 . 24885220 10.1186/1745-6215-15-170 PMC4037747

[16] Freedman SB, Ali S, Oleszczuk M, Gouin S, Hartling L. Treatment of acute gastroenteritis in children: an overview of systematic reviews of interventions commonly used in developed countries. Evid Based Child Health. 2013;8(4):1123–37. doi: 10.1002/ebch.1932 . 23877938 10.1002/ebch.1932

[17] Urbanska M, Gieruszczak-Bialek D, Szajewska H. Systematic review with meta-analysis: Lactobacillus reuteri DSM 17938 for diarrhoeal diseases in children. Aliment Pharmacol Ther. 2016;43(10):1025–34. doi: 10.1111/apt.13590 . 26991503 10.1111/apt.13590

[18] Victora CG, Adair L, Fall C, Hallal PC, Martorell R, Richter L, et al Maternal and child undernutrition: consequences for adult health and human capital. Lancet. 2008;371(9609):340–57. Epub 2008/01/22. doi: 10.1016/S0140-6736(07)61692-4 . 18206223 10.1016/S0140-6736(07)61692-4 PMC2258311

[19] Fischer Walker CL, Lamberti L, Adair L, Guerrant RL, Lescano AG, Martorell R, et al Does childhood diarrhea influence cognition beyond the diarrhea-stunting pathway? PloS one. 2012;7(10):e47908 doi: 10.1371/journal.pone.0047908 . 23118906 10.1371/journal.pone.0047908 PMC3485308

[20] Harris PA, Taylor R, Thielke R, Payne J, Gonzalez N, Conde JG. Research electronic data capture (REDCap)—a metadata-driven methodology and workflow process for providing translational research informatics support. J Biomed Inform. 2009;42(2):377–81. doi: 10.1016/j.jbi.2008.08.010 . 18929686 10.1016/j.jbi.2008.08.010 PMC2700030

[21] Ruuska T, Vesikari T. Rotavirus disease in Finnish children: use of numerical scores for clinical severity of diarrhoeal episodes. Scandinavian journal of infectious diseases. 1990;22(3):259–67. Epub 1990/01/01. doi: 10.3109/00365549009027046 . 2371542 10.3109/00365549009027046

[22] Gutierrez-Castrellon P, Lopez-Velazquez G, Diaz-Garcia L, Jimenez-Gutierrez C, Mancilla-Ramirez J, Estevez-Jimenez J, et al Diarrhea in preschool children and Lactobacillus reuteri: a randomized controlled trial. Pediatrics. 2014;133(4):e904–9. doi: 10.1542/peds.2013-0652 . 24639271 10.1542/peds.2013-0652

[23] Agustina R, Bovee-Oudenhoven IM, Lukito W, Fahmida U, van de Rest O, Zimmermann MB, et al Probiotics Lactobacillus reuteri DSM 17938 and Lactobacillus casei CRL 431 modestly increase growth, but not iron and zinc status, among Indonesian children aged 1–6 years. The Journal of nutrition. 2013;143(7):1184–93. doi: 10.3945/jn.112.166397 . 23700339 10.3945/jn.112.166397

[24] Goldfarb DM, Steenhoff AP, Pernica JM, Chong S, Luinstra K, Mokomane M, et al Evaluation of Anatomically-Designed Flocked Rectal Swabs for the Molecular Detection of Enteric Pathogens in Children Admitted to Hospital with Severe Gastroenteritis in Botswana. Journal of clinical microbiology. 2014 doi: 10.1128/JCM.01894-14 . 25165077 10.1128/JCM.01894-14 PMC4313226

[25] Mokomane M, Kasvosve I, Gaseitsiwe S, Steenhoff AP, Pernica JM, Lechiile K, et al A comparison of flocked swabs and traditional swabs, using multiplex real-time PCR for detection of common gastroenteritis pathogens in Botswana. Diagnostic microbiology and infectious disease. 2016;86(2):141–3. doi: 10.1016/j.diagmicrobio.2016.07.007 . 27460427 10.1016/j.diagmicrobio.2016.07.007 PMC5028275

[26] Kosek M, Haque R, Lima A, Babji S, Shrestha S, Qureshi S, et al Fecal Markers of Intestinal Inflammation and Permeability Associated with the Subsequent Acquisition of Linear Growth Deficits in Infants. The American journal of tropical medicine and hygiene. 2013;88(2):390–6. Epub 2012/11/28. doi: 10.4269/ajtmh.2012.12-0549 . 23185075 10.4269/ajtmh.2012.12-0549 PMC3583335

[27] Liu L, Oza S, Hogan D, Perin J, Rudan I, Lawn JE, et al Global, regional, and national causes of child mortality in 2000–13, with projections to inform post-2015 priorities: an updated systematic analysis. Lancet. 2014 doi: 10.1016/S0140-6736(14)61698-6 . 25280870 10.1016/S0140-6736(14)61698-6

[28] Pernica JM, Steenhoff AP, Welch H, Mokomane M, Quaye I, Arscott-Mills T, et al Correlation of Clinical Outcomes With Multiplex Molecular Testing of Stool From Children Admitted to Hospital with Gastroenteritis in Botswana. J Pediatr Infect Dis Soc. 2015 doi: 10.1093/jpids/piv028 26407262 10.1093/jpids/piv028 PMC5125452

[29] Platts-Mills JA, Gratz J, Mduma E, Svensen E, Amour C, Liu J, et al Association between stool enteropathogen quantity and disease in Tanzanian children using TaqMan array cards: a nested case-control study. The American journal of tropical medicine and hygiene. 2014;90(1):133–8. Epub 2013/11/06. doi: 10.4269/ajtmh.13-0439 . 24189366 10.4269/ajtmh.13-0439 PMC3886409

[30] Gough EK, Moodie EE, Prendergast AJ, Johnson SM, Humphrey JH, Stoltzfus RJ, et al The impact of antibiotics on growth in children in low and middle income countries: systematic review and meta-analysis of randomised controlled trials. BMJ. 2014;348:g2267 doi: 10.1136/bmj.g2267 . 24735883 10.1136/bmj.g2267 PMC3988318

[31] Bassler D, Briel M, Montori VM, Lane M, Glasziou P, Zhou Q, et al Stopping randomized trials early for benefit and estimation of treatment effects: systematic review and meta-regression analysis. JAMA: the journal of the American Medical Association. 2010;303(12):1180–7. Epub 2010/03/25. doi: 10.1001/jama.2010.310 . 20332404 10.1001/jama.2010.310

[32] Traa BS, Walker CL, Munos M, Black RE. Antibiotics for the treatment of dysentery in children. International journal of epidemiology. 2010;39 Suppl 1:i70–4. doi: 10.1093/ije/dyq024 . 20348130 10.1093/ije/dyq024 PMC2845863

[33] Statement on Travellers' Diarrhea. In: (CATMAT) CtAoTMaT, editor.: Public Health Agency of Canada; 2015.

[34] Travellers' Diarrhea Atlanta, GA: Centers for Disease Conrol; 2015 [cited 2015 May 10]. http://wwwnc.cdc.gov/travel/yellowbook/2016/the-pre-travel-consultation/travelers-diarrhea.

[35] Guerrant RL, Bartelt LA, Scharf RJ. Thinking deeper about important mass treatment trials. Clinical infectious diseases: an official publication of the Infectious Diseases Society of America. 2012;54(11):1674–5. doi: 10.1093/cid/cis241 . 22431805 10.1093/cid/cis241 PMC3404721

[36] Oria RB, Murray-Kolb LE, Scharf RJ, Pendergast LL, Lang DR, Kolling GL, et al Early-life enteric infections: relation between chronic systemic inflammation and poor cognition in children. Nutrition reviews. 2016;74(6):374–86. doi: 10.1093/nutrit/nuw008 . 27142301 10.1093/nutrit/nuw008 PMC4892302

[37] Applegate JA, Fischer Walker CL, Ambikapathi R, Black RE. Systematic review of probiotics for the treatment of community-acquired acute diarrhea in children. BMC public health. 2013;13 Suppl 3:S16 . 24564646 10.1186/1471-2458-13-S3-S16 PMC3847198

[38] Roos S, Dicksved J, Tarasco V, Locatelli E, Ricceri F, Grandin U, et al 454 pyrosequencing analysis on faecal samples from a randomized DBPC trial of colicky infants treated with Lactobacillus reuteri DSM 17938. PloS one. 2013;8(2):e56710 doi: 10.1371/journal.pone.0056710 . 23468874 10.1371/journal.pone.0056710 PMC3585302

[39] Jones SE, Versalovic J. Probiotic Lactobacillus reuteri biofilms produce antimicrobial and anti-inflammatory factors. BMC Microbiol. 2009;9:35 doi: 10.1186/1471-2180-9-35 . 19210794 10.1186/1471-2180-9-35 PMC2653509

