# Correction: Rapid enteric testing to permit targeted antimicrobial therapy, with and without *Lactobacillus reuteri* probiotics, for paediatric acute diarrhoeal disease in Botswana: A pilot, randomized, factorial, controlled trial

**DOI:** 10.1371/journal.pone.0194957

**Published:** 2018-03-21

**Authors:** Jeffrey M. Pernica, Andrew P. Steenhoff, Margaret Mokomane, Banno Moorad, Kwana Lechiile, Marek Smieja, Loeto Mazhani, Ji Cheng, Matthew S. Kelly, Mark Loeb, Ketil Stordal, David M. Goldfarb

The dose of *Lactobacillus reuteri* DSM 17938 appears incorrectly throughout the article as 5x10^8^ colony-forming units (cfu) per day. The correct dose of of *Lactobacillus reuteri* DSM 17938 should be 1x10^8^ cfu per day.
