# Supplementary material for: Rapid enteric testing to permit targeted antimicrobial therapy, with and without Lactobacillus reuteri probiotics, for paediatric acute diarrhoeal disease in Botswana: A pilot, randomized, factorial, controlled trial
Source: PLoS One. 2017 Oct 9;12(10):e0185177. doi: 10.1371/journal.pone.0185177 (PMC5633142; doi:10.1371/journal.pone.0185177)
Supplement: S2 File — Original trial protocol. (DOCX) [file pone.0185177.s002.docx]

**RESEARCH PROPOSAL**

**1. THE NEED FOR A TRIAL**

**1.1 What is the problem to be addressed?**

Diarrhoeal disease is the second-leading cause of death for children under the age of five ([1](#_ENREF_1)). The majority of these deaths are in lower-income countries; mortality associated with gastroenteritis is rare in North America ([2](#_ENREF_2)). A recent major study including over five thousand children presenting to health centres in sub-Saharan Africa with moderate-to-severe diarrhoea documented an overall mortality of 3.2% ([3](#_ENREF_3)). Unfortunately, the impact of diarrhoea is not limited to mortality; gastroenteritis causes a staggering amount of morbidity as well. A multi-country analysis pooling data from nine large longitudinal studies in lower-income countries showed that both the cumulative diarrhoeal incidence (number of diarrhoeal episodes/year) and the longitudinal diarrhoea prevalence (proportion of days with diarrhoea) were both associated with stunting (height-for-age more than two standard deviations below the mean) ([4](#_ENREF_4)). With every five episodes of diarrhoea, a 13% increase in the odds of stunting at 2 years was observed; the proportion of stunting at age 2 attributable to 5+ diarrhoeal episodes was estimated at 25% ([4](#_ENREF_4)). Stunting, of course, is a critical measure of well-being, as height-for-age at 2 years has been shown to be the best predictor of human capital ([5](#_ENREF_5)). Enteric infections in infants and toddlers have also been associated with cognitive deficits, less attained schooling, less economic productivity, metabolic derangements, and, least surprisingly, lower adult height ([5-7](#_ENREF_5)).

Most episodes of diarrhoea in children are caused by enteric viral pathogens, which cannot be treated with antimicrobials; the essentials of management for patients with viral gastroenteritis are rehydration, zinc treatment, and correction of electrolyte imbalances([8](#_ENREF_8)). In an effort to prevent some of these episodes, rotavirus vaccine has been integrated into the universal vaccination programs of many lower-income countries within the past five years. However, acute gastroenteritis can also be caused by bacterial and protozoan pathogens, and these infections *can* be attenuated with prompt antimicrobial therapy. Though many of these infections will eventually spontaneously resolve ([9](#_ENREF_9)), it is the standard of care in Canada – where access to good medical care is free, where HIV and TB are very rare in children, and where severe malnutrition is essentially an anachronism – to treat enteric infections caused by *Shigella, Campylobacter,* and enterotoxigenic *E. coli* (ETEC) with antimicrobials. In sub-Saharan countries, where mortality from diarrhoea is orders of magnitude higher, it is only rational to recommend the same. There are no placebo-controlled randomized trials showing a mortality benefit from antimicrobial therapy for bacterial enteritis in lower-income countries, possibly due to the lack of clinical equipoise around this point, but a recent systematic review hypothesized that antimicrobial therapy leads to a 99% reduction in mortality from bacterial enteritis associated with bloody stools ([10](#_ENREF_10)). Much time and effort has been made into developing a vaccine against ETEC to prevent the mortality in young children presumed to be attributable to this pathogen ([11](#_ENREF_11)).

Unfortunately, clinicians in many parts of the world do not have ready access to rapid, reliable diagnostics that can provide a microbiologic (aetiologic) diagnosis for acute gastroenteritis. Consequently, cases of diarrhoea without blood are presumed to be viral, and current World Health Organization guidelines recommend against empiric antimicrobial therapy for cases of uncomplicated acute gastroenteritis([8](#_ENREF_8)). In contrast, if there is bloody stools (dysentery), the likelihood that the diarrhoea is caused by a treatable pathogen increases substantially; current WHO guidelines suggest empiric therapy for dysentery using antibacterials targeted against *Shigella* spp., widely assumed to be the most common severe cause of bacillary dysentery ([12](#_ENREF_12)). The problem with this approach is that in many regions the proportion of non-dysenteric diarrhoea caused by treatable bacteria and protozoa may be substantial. Our preliminary results show that in Gaborone, Botswana, in 2011, over 35% of over five hundred children admitted to hospital because of gastroenteritis – most of whom did not have dysentery – were found to be harbouring a treatable enteric pathogen using advanced molecular diagnostic techniques.

All agree that bacillary (or protozoan) dysentery should be treated with antibiotics – what about bacillary (or protozoan) gastroenteritis not accompanied by bloody stools? The Global Enteric Multi-Center study found that two of the pathogens most closely linked with deaths associated with acute gastroenteritis were ETEC and *Cryptosporidium*, neither of which cause dysentery ([13](#_ENREF_13)). It is the standard of care in North America to treat traveller’s diarrhoea (most commonly caused by ETEC) with antibiotics to reduce morbidity; a Cochrane review has provided the evidence base for this practice ([14](#_ENREF_14)). ETEC, of course, has been hypothesized to be the most common cause of bacterial enteritis in young children in lower-income countries and is likely to cause much more severe disease in this population compared to healthy adult travellers from high-income countries ([11](#_ENREF_11)). Even pathogens that can cause dysentery are often treated with antimicrobials when bloody stools are not present; in a randomized control trial (RCT) investigating optimal therapy for shigellosis in Israel, 40% of study participants did not have bloody diarrhoea ([15](#_ENREF_15)). A randomized placebo-controlled trial examining antibiotic therapy for campylobacteriosis in Croatia, where none of the 120 participants had severe dehydration (in contrast to patients typically hospitalized for acute gastroenteritis in Africa), included only 10 participants with visibly bloody stools. Despite the fact that few subjects had dysentery, azithromycin was shown to lead to significantly increased rates of clinical cure as compared to placebo, as well as significantly faster improvement ([16](#_ENREF_16)). Leading researchers in global child health have suggested that important health outcome improvements could be seen with even minor interventions targeted at treatable bacterial and protozoan enteric infections; the context for this statement was the observation of major reductions in all-cause mortality after five thousand Ethiopian children were given one dose of azithromycin in a mass treatment trial for trachoma, a large proportion of which presumably did not have acute severe dysentery at the time ([17](#_ENREF_17)). Overall, it would seem logical that children with proven infectious bacterial/protozoan diarrhoea would benefit from antimicrobial therapy regardless of whether they have blood in their stool.

To permit timely treatment of bacterial/protozoan gastroenteritis, rapid diagnosis is needed (empiric treatment of all children with gastroenteritis with antibacterials and antiparasitics would be ill-advised from both medical and economic standpoints). Unfortunately, standard microbiologic techniques for the diagnosis of bacterial enteritis are labour-intensive, cannot detect ETEC, and require special equipment for the detection of *Campylobacter*; they also take 2-3 days in highly resourced laboratories, to say nothing of typical laboratories in sub-Saharan Africa. Consequently, advanced diagnostics – molecular diagnostics or simple immunoassays – are needed. Once these diagnostics have been integrated into routine clinical care of young children in lower-income-country settings, rapid treatment will become possible, and the mortality and morbidity benefits can be directly measured. This evaluation will be critical in determining how many children’s lives will be saved, and how much child stunting can be prevented, by their integration into routine care.

Probiotic therapy – the administration of ‘good’ bacteria – also holds promise for the supportive treatment of children with infectious gastroenteritis. A Cochrane review pooled data from 63 studies of proven or presumed infectious acute diarrhoeal disease (n=8014) and found that probiotic treatment decreased duration of diarrhoea by a mean of 24.5 hours (95% CI 16-34 h) and reduced stool frequency on day 2 of illness by a mean of 0.8 stools (95% 0.5-1.1 stools) in infants and young children, though many different probiotic preparations were used ([18](#_ENREF_18)). Almost as importantly, no significant or serious adverse reactions were found in all of these children. The majority of these studies were done in children with viral gastroenteritis, and there is no substantial evidence supporting the use of probiotics in children with bacterial or protozoal enteritis; however, it must be noted that these studies did not use advanced molecular diagnostics, and so it is very possible that many of these children had unrecognized bacterial co-infections. Another Cochrane systematic review has demonstrated that probiotic therapies can prevent paediatric antibiotic-associated diarrhoea when administered concurrently with antibiotics, suggesting that concomitant administration of antibacterials will not necessarily render the probiotics inactive ([19](#_ENREF_19)). Overall, there is a rational basis for exploring the use of probiotics in the sub-Saharan context for the treatment of infectious enteritis, whether viral, bacterial or protozoan.

**1.2 What are the principal research questions to be addressed?**

Our research question is whether, in children under five years of age admitted to hospital because of acute non-dysenteric diarrhoeal disease, rapid testing of stools for treatable bacterial/parasitic pathogens – accompanied by prompt treatment of these pathogens if detected – plus treatment with *Lactobacillus GG***,** will lead to decreased mortality and stunting at 60 days. Testing this hypothesis will likely require a large multicentre randomized trial, so we propose to first conduct a **pilot** **whose goal will be to demonstrate feasibility**. The specific feasibility criteria of this pilot are:

1) to enroll at least 100 participants during the 12-month study period;

2) to ensure that >95% of participants have a rectal swab taken within 18 hours of admission;

3) to verify that >95% of rectal swabs in the experimental group are resulted within 48 hours of admission;

4) to ensure >95% of participants found to have a treatable enteric pathogen are prescribed appropriate antimicrobials;

5) to ensure that >95% of participants prescribed antimicrobials start them within 24 hours of the test being resulted;

6) to contact >95% of participants 7-14 days after discharge from hospital; and

7) to contact >90% of participants 60 days after admission to hospital.

All aspects of the proposed follow-up trial will be assessed, including enrolment, randomization, follow up, methods for outcome assessment, and event rates. The data collection forms and data management strategy will be tested, data to derive a precise sample size will be generated, and assumptions for a cost effectiveness analysis will be assessed.

**1.3 Why is a pilot study necessary?**

This proposed pilot study is an essential first step prior to conducting an expensive multicentre trial. The process of the study must be verified; we will need to optimize communication between the study nurse and the microbiology laboratory to ensure that study specimens are obtained in a timely fashion, processed completely, and that results are communicated in an efficient manner back to the treating physicians. We will also need to ensure that a mechanism is put in place to ensure follow-up and that all outcomes can be reliably measured. We will likely need to compare various point-of-care assays to determine which will perform optimally in a clinical trial setting.

**1.4 How will the results of the trial be used?**

Before the widespread introduction of a new diagnostic test, there must be demonstrable benefit associated with its use; this is even more important in a setting such as sub-Saharan Africa where the public health care systems have very limited resources. Many organizations, both public and private, are spending time developing novel diagnostics – but no one has yet investigated their use in a systematic fashion within the parameters of a randomized controlled trial. The results of this trial will be of paramount importance to clinicians, patients, and health-care policy-makers in resource-limited settings.

**2. THE PROPOSED TRIAL**

**2.1 What is the proposed trial design?**

A single-centre, randomized, factorial (2x2), controlled, pilot trial. Children aged 3-60 months admitted to the paediatric ward of the Princess Marina Hospital in Gaborone, Botswana, with acute non-dysenteric diarrhoeal disease will be randomized to one of four arms: rapid stool testing/targeted antimicrobial therapy plus probiotic treatment, rapid stool testing/targeted antimicrobial therapy plus placebo treatment, delayed stool testing plus probiotic treatment, or delayed stool testing plus placebo treatment. The primary outcome is 60-day mortality. See Appendix 1 for flowchart of study procedures.

**2.2 What is the population under study?**

**2.2.1 Inclusion criteria.**

Children aged 3 to 60 months admitted to the Princess Marina Hospital paediatric medical ward primarily because of acute diarrhoeal disease will be eligible. Acute diarrhoeal disease will be defined as at least three watery stools in the 24 hours preceding admission to hospital. Children with HIV infection will not be excluded. Children with severe acute malnutrition will be eligible to be included in the rapid vs delayed testing and targeted treatment arms, but will not be randomized to probiotic (or placebo).

**2.2.2 Exclusion criteria.**

Children will be excluded if they have any of the following:

a) visibly bloody diarrhoea by history or at presentation, or

b) diarrhoea for > 14 days prior to admission to hospital, or

c) known inflammatory bowel disease, cystic fibrosis, or malignancy, or

d) suspected bacterial sepsis or meningitis leading to the initiation of ceftriaxone, or

e) severe metabolic acidosis with pH<7.1 or HCO3 < 10, or

f) suspected primary urinary tract infection and a urinalysis showing pyuria or positive nitrites, or

g) suspected primary diagnosis of pneumonia with a chest x-ray showing a focal consolidative process, or

h) live in a household with another individual documented to have an enteric infection of defined aetiology, or

i) are transferred from another health centre with treatment already begun with nalidixic acid, cefotaxime, or ciprofloxacin.

Children will also be excluded if they live outside Gaborone/Tlokweng/Mmopane, if they do not have a permanent address, and if a caregiver does not have ready access to a telephone (landline or mobile). We will not be enrolling children admitted to hospital for another reason who develop nosocomial diarrhoea > 48 h subsequent to enrollment. Children who require admission to hospital because of acute diarrhoeal disease who previously had been participants will not be eligible for participation again.

**2.3 What are the planned trial interventions?**

**2.3.1 All participants.** Data to generate Vesikari scores([20](#_ENREF_20)), a validated measure of gastroenteritis severity, will be collected at enrollment, and all study participants will be weighed and measured using calibrated apparatus. Percent dehydration will be abstracted from the paediatric team admission note (subjective dehydration) and will also be calculated comparing weight nadir in hospital to weight at discharge from hospital (objective dehydration). All participants will have a rectal flocked swab taken at enrollment within 18 hours of admission. Most of these children will not be started on antimicrobials at admission, as per current Princess Marina Hospital and World Health Organization standards, excepting children with severe acute malnutrition. Rehydration procedures will be decided by the attending physician; as per Princess Marina Hospital dehydration protocols, all those with severe dehydration receive intravenous rehydration with isotonic fluids and those with mild-moderate dehydration receive WHO-standard oral rehydration solution (ORS). It is standard of care at Princess Marina Hospital to also provide zinc treatment for gastroenteritis.

**2.3.2 Rapid testing and targeted antimicrobial therapy plus probiotic therapy arm.** These participants will be started on *Lactobacillus reuteri* within 24 hours of enrollment**.** All of these participants’ rectal swabs will be processed using our novel diagnostic techniques and resulted within 48 hours. It will be recommended to the attending physician that *Shigella,* ETEC, and *Campylobacter* infections be treated with azithromycin, and *Cryptosporidium* infections be treated with nitazoxanide. It will also be recommended that antimicrobials will be started as soon as possible, preferably on the day the test results become available, and antimicrobials will be provided free of charge. Probiotic therapy will be continued until the measurement of the primary outcome, 2 months post-enrollment.

**2.3.3 Rapid testing and targeted antimicrobial therapy plus placebo therapy arm.** These participants will be started on placebo therapy within 24 hours of enrollment and will have rectal swabs processed rapidly (see 2.3.2). Placebo therapy will be continued until the measurement of the primary outcome, 2 months post-enrollment.

**2.3.3 Delayed swab testing plus probiotic therapy arm.** These participants will be started on *Lactobacillus reuteri* within 24 hours of enrollment. Rectal swabs taken from children in the ‘delayed swab result’ arms will be batched and processed after all enrollment has been completed, and management in hospital will be at the discretion of the attending physician following Princess Marina Hospital standards of care. Should the attending physician consider the diagnosis of bacterial enteritis or cryptosporidiosis based on clinical judgment, azithromycin and/or nitazoxanide will be made available. Probiotic therapy will be continued until the measurement of the primary outcome, 2 months post-enrollment.

**2.3.4 Delayed swab testing plus placebo therapy arm.** These participants will be started on placebo therapy within 24 hours of enrollment and will have rectal swabs resulted only after the conclusion of the study (see 2.3.3). Should the attending physician consider the diagnosis of bacterial enteritis or cryptosporidiosis based on clinical judgment, azithromycin and/or nitazoxanide will be made available. Placebo therapy will be continued until the measurement of the primary outcome, 2 months post-enrollment.

**2.4 Laboratory testing.** A flocked rectal swab (Copan Italia S.P.A, Brescia, Italy) will be used to acquire specimens from all study participants; these will be placed in vials containing ENAT medium (Copan Italia S.P.A.) for stabilization and transport. A stool sample will be taken from each participant (as soon as reasonably possible) and stored at -70C at the National Microbiology Laboratory; these will then be batched and sent to McMaster for assessment of faecal calprotectin levels. An aliquot of the ENAT medium will have DNA extracted using QIAgen total nucleic acid extraction columns and the extracted DNA will be subjected to amplification and detection using multiplex taqman assays targeting *Shigella* spp., *Campylobacter* spp., *Salmonella* spp., ETEC and *Cryptosporidium* spp. previously adapted by our group for use on the ABI 7500 Realtime FAST qPCR platform. The remainder of laboratory testing that is required (eg. verification of HIV status, if applicable) will be done as per current Princess Marina Hospital standards of care. A stool sample will be obtained at the 2-month follow-up visit that will be sent to McMaster for faecal enzyme measurement and calculation of the environmental enteropathy score (composite of faecal alpha-1 antitrypsin, neopterin, and myeloperoxidase).

**2.5 What are the proposed practical arrangements for allocating participants to trial groups?**

A statistician independent of the study will generate a randomization list such that participants can be assigned at random to one of the four study groups in a 1:1:1:1 ratio. The randomization code will be generated by a random number generator and will be blocked (randomly in groups of 4-8), with the random blocking not disclosed. Given the substantial increase in mortality among children with severe acute malnutrition, randomization will be stratified by its presence or absence. Randomization will be communicated to the research assistant using an internet-based system.

**2.6 What are the proposed methods for protecting against other sources of bias?**

To reduce the possibility of selection bias, all children of the appropriate age with the primary diagnosis of acute gastroenteritis will be approached to participate. There will be some selection bias in that the most ill children may die in the first few hours after admission (before enrollment), and these children might have a higher probability of having a treatable enteric infection; however, many of these children would probably be judged as potentially having ‘bacterial sepsis’ and therefore would not be eligible for enrollment anyway. Allocation concealment will be centralized using the web-based system. Loss to follow-up will be minimized by ensuring that all potential participants live locally, have a means of getting in contact, and are aware of follow-up procedures. Observer/interviewer bias at the time of outcome measurement will be minimized by precise standardization of measurement protocols; in any case, all of the important outcomes (mortality and growth) are objective measures, and therefore less susceptible to observer bias.

**2.7 What is the proposed frequency and duration of follow-up?**

The research assistant will contact the participant 7-14 days after discharge from hospital to ensure that the caregivers remember the timing of the second follow-up visit and to answer any relevant questions about the study. The research assistant will then contact the participant again 60 days after enrollment to assess the primary outcome and weigh and measure the participant, if applicable. It would be optimal to have another follow-up visit approximately 12 months after enrollment; this may not be feasible with the funds we have acquired for this project, but we will ask participants for consent to approach them should moneys become available.

**2.8 What are the proposed *clinical* outcome measures?**

**2.8.1 Primary clinical outcome.** The primary outcome is mortality. If the participant dies prior to the 60-day followup, the research assistant will determine the date of death as accurately as possible.

**2.8.2 Other clinical outcomes.** Secondary outcomes will include the following: weight z-score at 60-day followup adjusted for initial weight; height z-score at 60-day followup adjusted for initial height; presence of stunting at 60-day followup; length of stay in hospital; number of days with at least three watery stools; number of days of fever in hospital; 7-day mortality; recurrence of acute diarrhoea during the 60-day followup period; re-presentation to a medical professional because of acute diarrhoea during the 60-day followup period; readmission to a paediatric medical ward during the 60-day followup period; HIV viral load (detectable/undetectable) at the next assessment within 6 months of enrollment, for those participants that are HIV positive; and environmental enteropathy score([21](#_ENREF_21)) (composite of faecal alpha-1 antitrypsin, neopterin, and myeloperoxidase) at 60 days.

**2.9 How will the *clinical* outcome measures be measured at follow up?**

**2.9.1 Mortality.** Mortality will be ascertained by the research assistant, whether in hospital or after discharge.

**2.9.2 Secondary clinical outcomes**. Data related to secondary clinical outcomes associated with the participant’s stay in hospital (number of days of 3+ watery stools, number of days of fever, length of stay) will be abstracted from the patient chart (both physician and nursing notes) at discharge. The remaining secondary clinical outcomes will be assessed at the 60-day followup visit; the research assistant will use a calibrated scale and stadiometer to obtain precise anthropomorphic measures.

**2.10 What are the *feasibility* outcomes, or criteria for success of this pilot study?**

The specific feasibility criteria of this pilot are:

1) to enroll at least 100 participants during the 12-month study period;

2) to ensure that >95% of participants have a rectal swab taken within 18 hours of admission;

3) to verify that >95% of rectal swabs in the experimental group are resulted within 48 hours of admission;

4) to ensure >95% of participants found to have a treatable enteric pathogen are prescribed appropriate antimicrobials;

5) to ensure that >95% of participants prescribed antimicrobials start them within 24 hours of the test being resulted;

6) to contact >95% of participants 7-14 days after discharge from hospital; and

7) to contact >90% of participants 60 days after admission to hospital.

**2.11 What is the proposed sample size?**

For this pilot study, a sample of 100 participants will be adequate to evaluate the feasibility criteria.

**2.12 What is the planned recruitment rate? What is the likely loss to follow-up?**

We are currently refining our novel enteric diagnostics and are enrolling an average of 4 children per day at Princess Marina Hospital, the majority of which would be eligible for the proposed pilot trial. Consequently, it should be possible to enroll all 100 participants within 9 months. In our other studies, we have had extremely good follow-up rates (1 of 60 loss to follow-up at 3 months post-enrollment), so we anticipate having very few (fewer than 5 participants) lost to follow-up in this project. We have budgeted to be able to visit families in their home should they be unable or insufficiently organized to return to Princess Marina Hospital for the final 2-month follow-up visit.

**2.13 What is the proposed type of analysis?**

The principal analysis will be intention-to-treat, i.e. all participants will be analyzed in the groups to which they were randomized, regardless if they received the appropriate antimicrobials (for those in the rapid swab analysis groups) or if they were adherent to the probiotic treatment (for those in the relevant groups). As the primary outcome (mortality) is binary, the chi-square test will be used. The chi-square test will also be used to interpret the following secondary outcomes: presence of stunting at 60-day followup, 7-day mortality, recurrence of acute diarrhoea during the 60-day followup period, re-presentation to a medical professional or readmission to Princess Marina Hospital because of acute diarrhoea in the 60-day followup period, and detectable HIV viral load within 6 months of enrollment. Analysis of covariance or logistic regression will be used to analyze the following secondary outcomes: height z-score at 60 days adjusted for initial height, weight z-score at 60 days adjusted for initial weight, and environmental enteropathy score at 60 days. Survival analysis will be used to analyze the following secondary outcomes: length of stay in hospital, number of days with at least three watery stools, and number of days of fever in hospital. Secondary analyses will attempt to control for the time to initiation of appropriate antimicrobial therapy and the time to initiation of probiotic therapy. Statistical analysis will be conducted using STATA 11.0 (College Station, TX).

**2.14 Will there be any sub-group analysis?**

Children with severe acute malnutrition are functionally significantly immune compromised, and are very different than those without; consequently, we will attempt to analyze this group separately, though definite conclusions will be difficult to make if numbers are small. Mortality in this sub-group is much higher than in well-nourished children, so even if the benefits of the experimental interventions are less, it is important to include them in this trial. We will also explore the impact of HIV infection/exposure and the severity of the initial presentation on the analysis. We should also note that preliminary data suggest that the benefit of probiotic therapy is much greater in children with viral gastroenteritis; consequently, we will be analyzing those separately from those with no detected viral infections.

**2.15 Potential problems and alternative strategies.**

Our biggest concern with this study is avoiding loss to follow-up; this is why to be eligible the potential participant must reside within a 50 km radius of Princess Marina Hospital, be relatively stable, and have some means by which the research assistant can contact them. We will be providing simple incentives to encourage families to accept follow-up.

It will also be of prime importance to minimize the delay between rectal swab acquisition, finalizing the result, and initiating appropriate antimicrobial therapy (if applicable) in the rapid swab result groups. We are currently optimizing laboratory procedures and flow-through at the Botswana National Health Laboratory using already-developed molecular assays and an ABI 7500 platform. Should there be unacceptable delays in specimen processing, we will explore alternative platforms.

**2.16 Ethical considerations**

**2.16.1 Risks to the safety of participants involved in the trial.**

There should be very little risk to study participants. The use of the flocked rectal swab itself – the most invasive study procedure – should pose negligible risk; the process is very similar to that used for measuring rectal temperatures, and to date, we have acquired over 500 of these specimens without a single complication. The other risk associated with the study would be breach of confidentiality, and we will take all standard precautions to guard against that: the key document linking participant names, addresses, and other direct identifiers will be kept in a locked cabinet (and a password-protected computer) in a locked office to which only the local PI and/or study nurse will have access; the case report forms with study data will be labeled only with the study ID; and no information containing direct identifiers will ever be sent electronically. Stool samples labeled with study IDs (and no direct identifiers) will be sent to Canada for enzyme testing but no genetic testing will be done.

Probiotic therapy – the administration of viable ‘good’ bacteria to patients – is not the current standard of care in Botswana for the treatment of gastroenteritis. However, as detailed previously in this protocol, numerous studies done in both higher-income and lower-income countries have documented clinically significant improvements in presumed infectious gastroenteritis associated with probiotic use and no significant adverse reactions have been observed after treatment of thousands of immunocompetent children. Children with severe acute malnutrition are obviously not functionally immunocompetent, so they will not be eligible for probiotic treatment in this study.

It will be explained to participants that they have much to potentially gain by being in the study; should our testing detect a treatable pathogen, this information will be communicated to the attending physician in a timely manner. Furthermore, in many cases, the study will provide drug therapy free of charge to the study participant; azithromycin should be superior to nalidixic acid, currently the standard of care for the treatment of bacillary dysentery at Princess Marina Hospital.

**2.16.2 Informed consent**.

Informed consent will of course be a necessary prerequisite to enrollment in the study. The research nurse will explain the trial in detail on the paediatric medical ward at Princess Marina Hospital to the caregiver that is present with the child. As all of our patients will be younger than five years in age, and there are no real invasive procedures associated with the study protocol, we will not be seeking assent from the child participants. Consent will be sought for the repeat visits at 12 months post enrollment, and to analyze the bacterial populations (microbiome analysis) of the stool sent to McMaster University, even though those are not key aspects of the trial; we do not yet have funding for these components, but it would be most appropriate to ask for consent up front.

**3.0 TRIAL MANAGEMENT**

**3.1 Day-to-day management of the trial.**

The coordinating centre will be at the Botswana UPenn-Partnership. The local PI, Dr. Andrew Steenhoff, will have ongoing communication with the research nurse to monitor enrollment, verify thorough and complete data collection, and troubleshoot issues. All microbiology testing will take place at the National Health Laboratory (NHL) under the supervision of Mrs Margaret Mokomane. The study PI will arrange brief teleconferences with BUP and NHL personnel to monitor study progress on a weekly basis, or as frequently as is needed to ensure smooth functioning of the trial. Paper case report forms will be digitized on a regular basis by BUP support staff or by the research nurse, as needed. All forms containing direct identifiers will be maintained under lock and key or in a password-protected computer in a locked office, but other data will be transmitted to McMaster University using a REDCap database.

**3.2 Quality control and assurance.**

An audit process will be completed after each 25 participants enrolled by the research nurse, other research assistants, or investigators. 5 records (per each 25) will be selected randomly to verify the following indicators: eligibility, consent forms, signatures, timing of specimen collection, timing of results, timing of antimicrobial administration (if applicable), collection of data relating to outcomes at the time the record is verified, and missing data. At these times a formal review of the case report forms with the research nurse will be undertaken to determine if improvements need to be made. The health records department at Princess Marina Hospital will be contacted to verify on a monthly basis the number of patients of the appropriate age admitted with acute gastroenteritis.

**3. REFERENCES**

1. Jensen L (ed.) *The millennium development goals report 2010*, (United Nations Department of Economic and Social Affairs, 2010).

2. Long SS, Pickering LK, Prober CG. *Principles and practice of pediatric infectious diseases.*, (Elsevier, Philadelphia, 2008).

3. Kotloff KL, Nataro JP, Blackwelder WC*, et al.* Burden and aetiology of diarrhoeal disease in infants and young children in developing countries (the global enteric multicenter study, gems): A prospective, case-control study. *Lancet* 2013

4. Checkley W, Buckley G, Gilman RH*, et al.* Multi-country analysis of the effects of diarrhoea on childhood stunting. *Int J Epidemiol* 2008 37, 816-830

5. Victora CG, Adair L, Fall C*, et al.* Maternal and child undernutrition: Consequences for adult health and human capital. *Lancet* 2008 371, 340-357

6. Guerrant DI, Moore SR, Lima AA*, et al.* Association of early childhood diarrhea and cryptosporidiosis with impaired physical fitness and cognitive function four-seven years later in a poor urban community in northeast brazil. *Am J Trop Med Hyg* 1999 61, 707-713

7. Guerrant RL, Kosek M, Lima AA*, et al.* Updating the dalys for diarrhoeal disease. *Trends Parasitol* 2002 18, 191-193

8. *Recommendations for management of common childhood conditions. Evidence for technical update of pocket book recommendations.*, (World Health Organization, Switzerland, 2012).

9. Mandell GL, Bennett JE, Dolin R. *Principles and practice of infectious diseases*, (Churchill Livingstone Elsevier, Philadelphia, 2010).

10. Traa BS, Walker CL, Munos M*, et al.* Antibiotics for the treatment of dysentery in children. *Int J Epidemiol* 2010 39 Suppl 1, i70-74

11. Zhang W & Sack DA. Progress and hurdles in the development of vaccines against enterotoxigenic escherichia coli in humans. *Expert Rev Vaccines* 2012 11, 677-694

12. *Guidelines for the control of shigellosis, including epidemics due to shigella dysenteriae type 1*, (WHO Document Production Services, Geneva, Switzerland, 2005).

13. Levine M. The global enteric multi-center study (gems): Etiology and burden of moderate and severe diarrheal disease in africa and asia. in *Pediatric Academic Societies annual conference* (Boston, 2012).

14. De Bruyn G, Hahn S & Borwick A. Antibiotic treatment for travellers' diarrhoea. *Cochrane Database Syst Rev* 2000, CD002242

15. Ashkenazi S, Amir J, Waisman Y*, et al.* A randomized, double-blind study comparing cefixime and trimethoprim-sulfamethoxazole in the treatment of childhood shigellosis. *J Pediatr* 1993 123, 817-821

16. Vukelic D, Trkulja V & Salkovic-Petrisic M. Single oral dose of azithromycin versus 5 days of oral erythromycin or no antibiotic in treatment of campylobacter enterocolitis in children: A prospective randomized assessor-blind study. *J Pediatr Gastroenterol Nutr* 2010 50, 404-410

17. Guerrant RL, Bartelt LA & Scharf RJ. Thinking deeper about important mass treatment trials. *Clin Infect Dis* 2012 54, 1674-1675

18. Allen SJ, Martinez EG, Gregorio GV*, et al.* Probiotics for treating acute infectious diarrhoea. *Cochrane Database Syst Rev* 2010, CD003048

19. Johnston BC, Goldenberg JZ, Vandvik PO*, et al.* Probiotics for the prevention of pediatric antibiotic-associated diarrhea. *Cochrane Database Syst Rev* 2011, CD004827

20. Ruuska T & Vesikari T. Rotavirus disease in finnish children: Use of numerical scores for clinical severity of diarrhoeal episodes. *Scand J Infect Dis* 1990 22, 259-267

21. Kosek M, Haque R, Lima A*, et al.* Fecal markers of intestinal inflammation and permeability associated with the subsequent acquisition of linear growth deficits in infants. *Am J Trop Med Hyg* 2013 88, 390-396

**APPENDICES**

**Appendix 1. Flowchart of Study Procedures.**

**Appendix 3**
